# Effects of *rpl1001* Gene Deletion on Cell Division of Fission Yeast and Its Molecular Mechanism

**DOI:** 10.3390/cimb46030164

**Published:** 2024-03-18

**Authors:** Wen Yu, Rongmei Yuan, Mengnan Liu, Ke Liu, Xiang Ding, Yiling Hou

**Affiliations:** 1 Key Laboratory of Southwest China Wildlife Resources Conservation (Ministry of Education), College of Life Science, China West Normal University, Nanchong 637009, China; 17345345095@163.com (W.Y.); 18482189593@163.com (R.Y.); 15881382969@163.com (M.L.); 18728348818@163.com (K.L.); 2 College of Environmental Science and Engineering, China West Normal University, Nanchong 637009, China

**Keywords:** *Schizosaccharomyces pombe*, *rpl1001* gene, cell division, RNA-Seq, differential gene expression

## Abstract

The *rpl1001* gene encodes 60S ribosomal protein L10, which is involved in intracellular protein synthesis and cell growth. However, it is not yet known whether it is involved in the regulation of cell mitosis dynamics. This study focuses on the growth, spore production, cell morphology, the dynamics of microtubules, chromosomes, actin, myosin, and mitochondria of fission yeast (*Schizosaccharomyces pombe*) to investigate the impact of *rpl1001* deletion on cell mitosis. RNA-Seq and bioinformatics analyses were also used to reveal key genes, such as *hsp16*, *mfm1* and *isp3*, and proteasome pathways. The results showed that *rpl1001* deletion resulted in slow cell growth, abnormal spore production, altered cell morphology, and abnormal microtubule number and length during interphase. The cell dynamics of the *rpl1001Δ* strain showed that the formation of a monopolar spindle leads to abnormal chromosome segregation with increased rate of spindle elongation in anaphase of mitosis, decreased total time of division, prolonged formation time of actin and myosin loops, and increased expression of mitochondrial proteins. Analysis of the RNA-Seq sequencing results showed that the proteasome pathway, up-regulation of *isp3*, and down-regulation of *mfm1* and *mfm2* in the *rpl1001Δ* strain were the main factors underpinning the increased number of spore production. Also, in the *rpl1001Δ* strain, down-regulation of *dis1* caused the abnormal microtubule and chromosome dynamics, and down-regulation of *hsp16* and *pgk1* were the key genes affecting the delay of actin ring and myosin ring formation. This study reveals the effect and molecular mechanism of *rpl1001* gene deletion on cell division, which provides the scientific basis for further clarifying the function of the Rpl1001 protein in cell division.

## 1. Introduction

Ribosomal proteins are not only associated with protein translation in the cytoplasm but are also involved in other cellular processes. In yeast, loss of ribosomal protein L32-2 (Rpl32) causes a decrease in ribosome levels, triggering mutual recognition of mating proteins on the cell surface, affecting the MAPK pheromone response pathway, and regulating the process of sexual reproduction [1]. The 60S ribosomal protein L22 (Rpl22) is a key regulator of the transcription factor Ime1 and regulates the cellular transition from mitosis to meiosis [2]. Ribosomal protein S3 (Rps3) localizes to the spindle, binds to the spindle throughout mitosis, participates in microtubule polymerization and spindle formation, and influences mitosis [3]. The activation of ribosomal protein S6 (Rps6) is mediated via its phosphorylation by ribosomal protein S6 kinase (S6K) to form p-RpS6. It participates in the complex 1 (mTORC1) pathway, which regulates the stabilization of actin cytoskeletal organization in yeast and is involved in cell growth and cell cycle processes [4,5].

**Figure 1:**
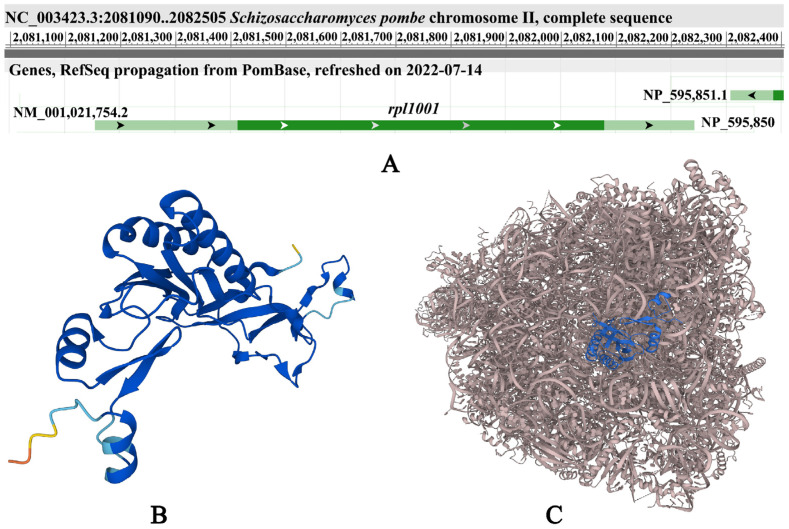
The *rpl1001* gene location and the Rpl1001 protein structure. (**A**) Location of the *rpl1001* gene. The figure is derived from the PomBase (https://www.pombase.org/, accessed on 28 November 2023). (**B**) Predicted structure of the Rpl1001 protein. (**C**) PDB structure of the Rpl1001 protein. The blue part shows the predicted structure of the Rpl1001 protein (PDB ID: 8EUI).

In fission yeast, the *rpl1001* gene encodes 60S ribosomal protein L10, a member of the L10e protein family (Figure 1). Within the ribosome, the Rpl1001 protein is located at the center of the topological connectivity of many functional regions in the large subunit, close to the GTPase center and the sarcin-ricin loop, and is almost completely covered by the small subunits in the 80S ribosome [6]. From yeast to humans, Rpl1001 is the main controller of ribosome structure and function, affecting key steps such as ribosome assembly and protein synthesis [7]. In the nucleolus of budding yeast, Rpl1001 is involved in the processing of pre-mRNA molecules [8], and in the cytoplasm, Rpl1001 participates in the ligation of the 40S and 60S ribosomal subunits [9]. In fission yeast, the deletion of *rpl1001* resulted in decreased cell growth in the media containing xylose or galactose as the carbon source and lysine, proline, or serine as the nitrogen sources, respectively, and increased sensitivity to cadmium, lithium, lithium chloride, magnesium chloride, methyl methanesulfonate, sodium dodecyl sulfate, tunicamycin, valproic acid, bleomycin, and vanadate [10]. In the absence of a nitrogen source, the cell cycle of *rpl1001Δ* cells arrests [11]. The *rpl1001* gene deficiency is closely linked to disease in humans. Rpl1001 is a zinc-binding regulatory protein which interacts with oncogenic transcription factor c-Jun, and inactivation of the Rpl1001 protein leads to nephroblastoma [12]. Missense mutations in *rpl1001* lead to translation defects, causing systemic central nervous system defects which affect brain formation [13]. Rpl1001 is also involved in the fine-tuning mechanism of synaptic plasticity, which is one of the central processes that are impaired in autism, and mutations in *rpl1001* gene usually lead to autism [14].

Cell division is essential to the survival of all organisms, and the process involves many structures working together, including the cytoskeleton, chromosomes, and mitochondria [15]. *Schizosaccharomyces pombe* (*S. pombe*) has a relatively simple and clear genetic system, a typical eukaryotic cell cycle, and a conserved division mechanism, making it a model organism suited for the study of cell division processes [16]. There is no information currently available on whether the *rpl1001* deletion affects mitotic kinetics and sexual reproduction. In our study, the *S. pombe* was used as the research model to investigate the effect of *rpl1001* gene deletion on the growth, spore production, and cell morphology and on the dynamics of microtubules, chromosomes, actin, myosin, and mitochondria. RNA-Seq and bioinformatics analyses were also used to reveal the key genes and pathways, which could provide a scientific foundation for further understanding the role of the Rpl1001 protein in cell division.

## 2. Materials and Methods

### 2.1. Experimental Strains

All strains used for the study are listed in Table 1.

### 2.2. Cell Growth Analysis

The PT287 and 2599-1 strains were cultured in YE5S solid medium at 25 °C for 48 h to reach the active state. The strains were transferred to YE5S liquid medium at 25 °C till the OD_595_ (optical density) reached 0.5~0.8, then the strains were diluted to OD_595_ = 0.1, and the OD_595_ values were measured continuously every 2 h for 12 h.

### 2.3. Spore Production Analysis

The h+ and h- strains were inoculated with YE5S solid medium and cultured at 25 °C for activation, and then the activated h+ and h- strains were mixed onto EMM-N medium at 25 °C for cultivating spores. After 48 h, the strains were picked for microscopic examination, and the spores produced were observed and counted. The total amount of cells evaluated in each group was 1500 [17].

### 2.4. Construction of Fluorescent Protein Tags

The *rpl1001Δ* (h+ and h-) strain and the wild-type (h+ and h-) strain with fluorescent protein marker were cultured to the activated state, and the activated h+ and h- strains were mixed onto EMM-N medium in 25 °C to cultivate spore production. After 48 h, yeast cells were subjected to snail enzyme (Shanghai Yuanye Bio-Technology Co., Ltd., Shanghai, China) treatment, the spores were removed and coated in YE5S solid medium for culture. The target strains were selected through resistance medium, nutrient-deficient medium, and microscopy.

### 2.5. Microscopy and Data Analysis

Live cell imaging was performed with a Leica TCS-SP8 (Leica Microsystems Ltd., Wetzlar, Germany) confocal microscope at 25 °C. In order to obtain high-quality images, the parameters during laser confocal imaging were set as follows: green fluorescence excitation wavelength was 488 nm, emission wavelength reception range of 491–549 nm, red fluorescence excitation wavelength was 552 nm, emission wavelength reception range of 583–635 nm, pixels of 512 × 512 μm, 7 optical sections of 1 µm distance, and an exposure time of 400 ms, which was acquired at 90 or 120 s intervals across a total time of 120 min. The program ImageJ was used to process the pictures. The software SPSS 17 was used for data analysis in the *rpl1001Δ* and wild-type strains. * *p* < 0.05 denotes a significant difference, ** *p* < 0.01 denotes a highly significant difference. The GraphPad Prism 8 (GraphPad Software Inc., La Jolla, CA, USA) was used to produce all of the box plot.

### 2.6. Bioinformatics Analysis

The 2599-1 and PT287 strains were cultured and shaken at 25 °C until they reached the logarithmic phase of cell division, then the strains were collected and frozen, and sent to Beijing Novogene Co., Ltd. (Beijing, China) for the extraction of RNA and quality testing. Agarose gel electrophoresis was used to analyze the degree of degradation of the RNA and the contamination status. NanoDrop (Thermo Fisher Scientific Inc., Waltham, MA, USA) was used to test the purity of the RNA. Qubit2.0 Fluorometer (Thermo Fisher Scientific Inc., Waltham, MA, USA) was used to accurately quantify the concentration of RNA. Agilent 2100 bioanalyzer (Agilent Technologies Inc., Santa Clara, CA, USA) was used to accurately detect the integrity of RNA. After the samples were tested and qualified, the cDNA library was constructed, and sequencing was performed after the library was qualified. Total RNA sequencing of the wild-type and *rpl1001Δ* strains was performed to obtain raw reads. Splice and low-quality data were removed to obtain clean reads, which were accurately compared with the reference genome by Hisat2 v2.0.5 software [18]. Quantitative analysis of gene expression was performed by the Feature Counts and Stringtie (1.3.3b) software [19,20], differential significance analysis of gene expression of the samples was performed by the DESeq2 (1.20.0) and EdgeR (3.22.5) software [21]. GO and KEEG pathway enrichment of differential genes were performed by the Cluster Profile (3.4.4) software [22]. The obtained transcriptome sequence was uploaded into NCBI with the access number PRJNA1046258.

### 2.7. Quantitative Real-Time PCR

Total RNA from logarithmic growth phase of yeast cell culture was extracted, reverse-transcribed to cDNA, and subjected to Quantitative Real-Time PCR (qRT-PCR) using the act1 gene as the reference gene to detect the gene expression level. The primers used are listed in Table 2.

## 3. Results

### 3.1. Changes of Cell Growth and Spore Production in the rpl1001Δ Strain

**Figure 2:**
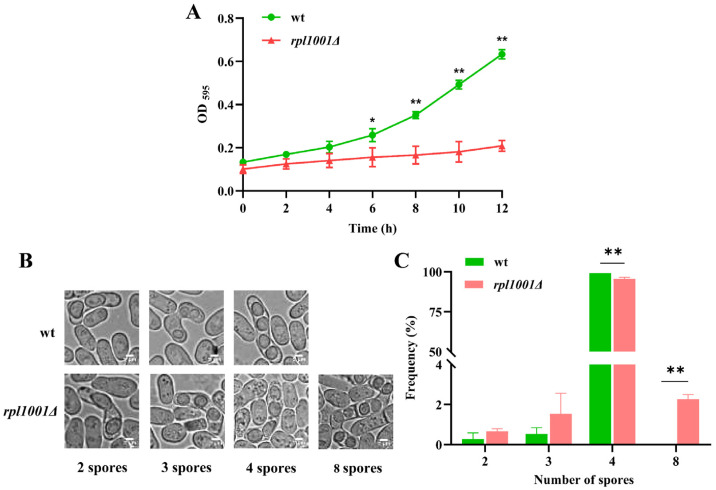
Changes of cell growth and spore production in the *rpl1001Δ* strain. (**A**) Growth curves of the wt and *rpl1001Δ* strains (*n* = 3). (**B**) Spore morphology in the wt and *rpl1001Δ* strains, scale bar: 2 μm. (**C**) Spore number analysis in the wt and *rpl1001Δ* strains (*n* = 1500). OD, optical density. * *p* < 0.05, ** *p* < 0.01.

The findings of cell proliferation showed that between 0 and 4 h, there was nearly no variation in the rates of proliferation between the wild-type and *rpl1001Δ* strains. However, after 4 h, the wild-type strain entered the logarithmic growth phase, while the *rpl1001Δ* strain showed obvious slow growth. At 12 h, the OD_595_ (optical density) of the wild-type strain was 0.7 and that of the *rpl1001Δ* strain was only 0.2, which was statistically significant (Figure 2A). The findings above showed that the *rpl1001Δ* strain became slower in growth.

A comparison of the number of spores generated by the wild-type and *rpl1001Δ* strains revealed that the former produced four spores at a rate of 99.2% and the latter at 95.5%. These differences were statistically significant. Additionally, 2.3% of the *rpl1001Δ* strains were found to produce eight spores (Figure 2B,C). The findings indicated an abnormal number of spore production in the *rpl1001Δ* strain, which tended to produce more than four spores.

### 3.2. Microtubule Alterations in the rpl1001Δ Strain during Interphase

**Figure 3:**
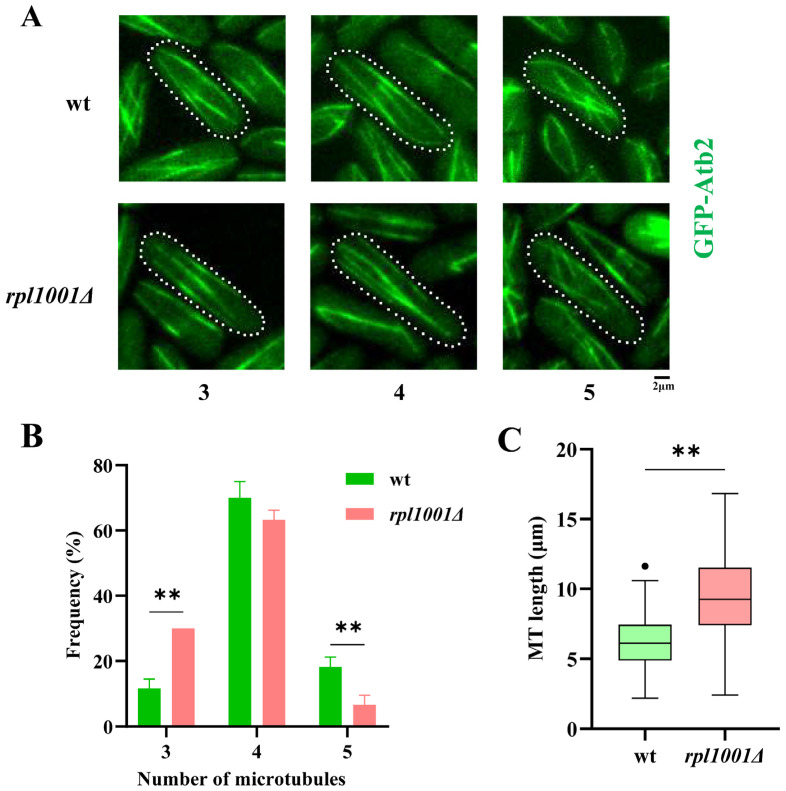
Changes in microtubules in the *rpl1001Δ* strain during interphase. (**A**) Morphological images of MTs in the wt and *rpl1001Δ* strains, white dotted lines indicate cell outlines. (**B**) Analysis of MT number in the wt and *rpl1001Δ* strains (*n* = 60). (**C**) Analysis of MT length in the wt and *rpl1001Δ* strains (*n* = 80), the black dot indicates outlier. GFP-Atb2, green fluorescent protein-tubulin α2. ** *p* < 0.01.

Analysis of microtubules (MTs) showed that the proportions of MT number in the wild-type and *rpl1001Δ* strains having 3, 4 and 5 MTs were 11.7 ± 2.9%, 70.0 ± 5.0%, 18.3 ± 2.9% and 30.0 ± 10.0%, 63.3 ± 2.9%, 6.7 ± 2.9%, respectively (Figure 3A). The number of cells with 3 and 5 MTs varied significantly between the *rpl1001Δ* and wild-type strains (Figure 3B). The average length of MTs of the *rpl1001Δ* strain was 9.3 ± 3.0 μm, which was significantly different from the wild-type (Figure 3C). In summary, the number of MTs decreased and the length increased in the *rpl1001Δ* strain during the interphase.

### 3.3. Changes in Chromosome and Spindle in the rpl1001Δ Strain during Mitosis

**Figure 4:**
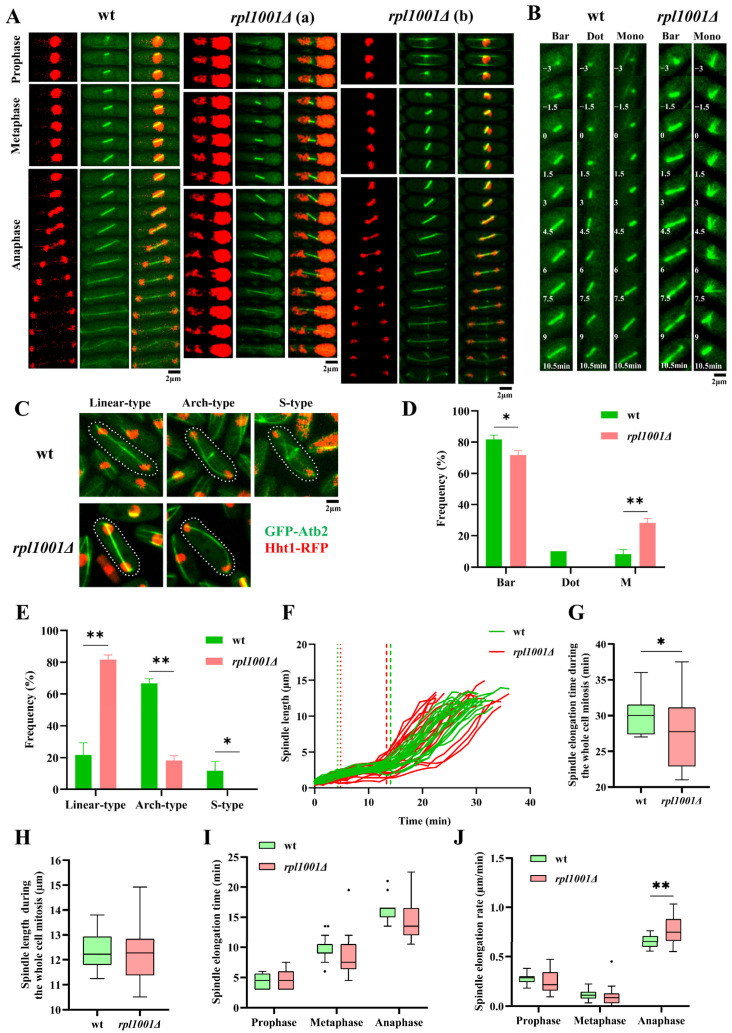
Changes of chromosome and spindle in the *rpl1001Δ* strain during mitosis. (**A**) Time-lapse images of chromosome and spindle in the wt and *rpl1001Δ* strains during whole mitosis. Each frame corresponded to a 90 s interval, *rpl1001Δ* (a) showed chromosome fragmentation and uneven segregation, *rpl1001Δ* (b) showed normal chromosomal segregation. (**B**) Formation type of spindle morphology in the wt and *rpl1001Δ* strains; 0 min indicates complete disintegration of interphase MTs. (**C**) Morphology of the wt and *rpl1001Δ* strains spindle breakage types, white dotted lines indicate cell outlines. (**D**) Spindle formation mode statistics in the wt and *rpl1001Δ* strains (*n* = 60). (**E**) Spindle breakage mode statistics in the wt and *rpl1001Δ* strains (*n* = 60). (**F**) Spindle elongation curve in the wt and *rpl1001Δ* strains during mitosis (*n* = 20), dotted lines indicate transition from prophase to metaphase, short linear lines indicate transition from metaphase to anaphase. (**G**) The total time from formation to breakage of the spindle in the wt and *rpl1001Δ* strains (*n* = 20). (**H**) Final length of spindle in the wt and *rpl1001Δ* strains (*n* = 20). (**I**) Spindle elongation time of the wt and *rpl1001Δ* strains (*n* = 20). (**J**) Spindle elongation rate in the wt and *rpl1001Δ* strains (*n* = 20). The black dots indicate outliers. GFP-Atb2, green fluorescent protein-tubulin α2; Hht1-RFP, histone H3-red fluorescent protein. * *p* < 0.05, ** *p* < 0.01.

During mitosis, MTs assemble into spindles in anaphase to help chromosomes segregate correctly. The results showed that 10.0% of cells of the *rpl1001Δ* strain had defects of chromosome fragmentation and uneven segregation (Figure 4A). Statistics on the spindle architecture revealed that 81.7 ± 2.9% of the wild-type strain spindles formed a bar, 10.0% formed a dot, and 8.3% formed a monopolar body with a transient protrusion of MT bundles. While in the *rpl1001Δ* strain, no dot spindle formation was found and the proportion of bar and monopolar spindle formation were 71.7 ± 2.9% and 28.3 ± 2.9%, respectively, indicating that the bipolar spindle was significantly reduced in the *rpl1001Δ* strain, and unipolar spindle was significantly increased (Figure 4B,D). The mode of spindle depolymerization was also measured, and the proportion of cells in which the spindle of the wild-type strain broke in the linear-type, arch-type, and S-type were 21.7 ± 7.6%, 66.7 ± 2.9%, and 11.7 ± 5.8%, respectively. The *rpl1001Δ* strain spindle had no S-type breaks, and the proportions of cells with the linear-type and arch-type breaks were 81.7 ± 2.9% and 18.3 ± 2.9%, respectively, which indicated that the *rpl1001Δ* strain had a highly significant increase in linear-type breaks (Figure 4C,E). In conclusion, the spindle formation and breakage pattern processes were abnormal in the *rpl1001Δ* strain.

The results of spindle dynamics showed that the total time from the forming of spindles to breakage in the wild-type and *rpl1001Δ* strains were 30.2 ± 2.5 and 27.8 ± 4.7 min, respectively. There was no difference in the final spindle lengths, which were 12.3 ± 0.7 and 12.2 ± 1.1 μm in the wild-type and *rpl1001Δ* strains, respectively (Figure 4F–H). The above results suggested that *rpl1001* gene deletion shortens the elongation time of the spindle but did not affect the final length of the spindle.

Further statistics on the kinetics of the spindle at different phases of mitosis showed that the times of spindle elongation at prophase, metaphase, and anaphase in the wild-type and *rpl1001Δ* strains were 4.3 ± 1.2, 9.8 ± 1.9, 16.1 ± 1.8 and 4.8 ± 1.7, 8.6 ± 3.6, 14.5 ± 3.4 min, respectively, and the spindle elongation rates at prophase and metaphase of the wild-type and *rpl1001Δ* strains were 0.3 ± 0.1, 0.1 ± 0.1 and 0.2 ± 0.1, 0.1 ± 0.1 μm/min, respectively. There were no significant difference between these two groups (Figure 4I,J). However, at anaphase, the spindle elongation rates of the wild-type and *rpl1001Δ* strains were 0.6 ± 0.1 and 0.8 ± 0.1 μm/min, respectively, showing significant differences (Figure 4J). These findings demonstrated that during anaphase, the elongation rate of the spindle was accelerated in the *rpl1001Δ* strain.

### 3.4. Changes of Cell Morphology in the rpl1001Δ Strain

**Figure 5:**
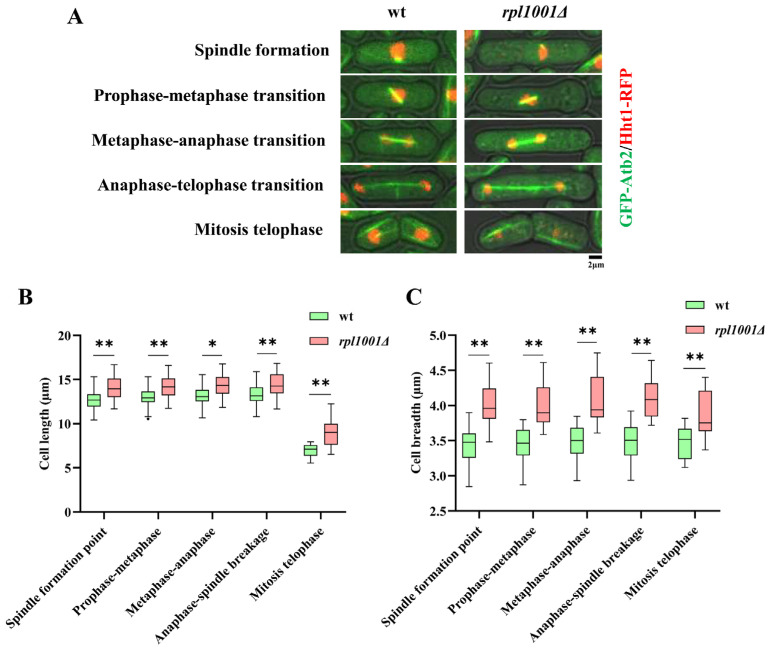
Changes of cell morphology in the *rpl1001Δ* strain. (**A**) Cell morphology of different phases in the wt and *rpl1001Δ* strains during mitosis. (**B**) Cell length of different phases in the wt and *rpl1001Δ* strains during mitosis (*n* = 20), the black dot indicates outlier. (**C**) Cell breadth of different phases in the wt and *rpl1001Δ* strains during mitosis (*n* = 20). GFP-Atb2, green fluorescent protein-tubulin α2; Hht1-RFP, histone H3-red fluorescent protein. * *p* < 0.05, ** *p* < 0.01.

The cell lengths at the spindle formation during prophase-metaphase, metaphase-anaphase, anaphase-telophase transitions and telophase of the wild-type strains were 12.8 ± 1.2, 13.0 ± 1.2, 13.1 ± 1.2, 13.3 ± 1.3, and 7.0 ± 0.8 μm, respectively, and those in the *rpl1001Δ* strains were 14.1 ± 1.4, 14.2 ± 1.4,14.3 ± 1.4, 14.3 ± 1.5, and 9.0 ± 0.6 μm, respectively (Figure 5A,B). The cell length of the *rpl1001Δ* strain was significantly different compared to the metaphase-anaphase transition, and the cell length of the *rpl1001Δ* strain was significantly increased at all other periods.

The cell breadth in each period of the wild-type strains was 3.5 ± 0.2, 3.5 ± 0.2, 3.5 ± 0.2, 3.5 ± 0.3, and 3.5 ± 0.2 μm, respectively, while for the *rpl1001Δ* strains they were 4.0 ± 0.3, 4.0 ± 0.3, 4.0 ± 0.3, 4.1 ± 0.3, and 3.9 ± 0.3 μm (Figure 5C), indicating that the *rpl1001Δ* strain also exhibited a highly significant increase in cell breadth in each period. In summary, the *rpl1001Δ* cells become longer and wider in all phases of mitosis, and the overall volume of the cells also increased.

### 3.5. Changes of the Actin and Myosin Ring in the rpl1001Δ Strain during Mitosis

**Figure 6:**
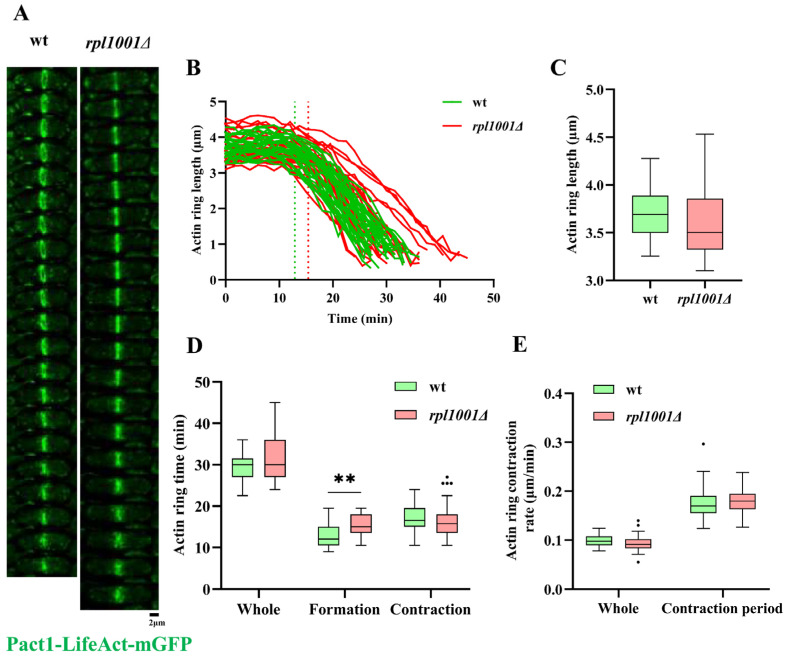
Changes in the actin ring in the *rpl1001Δ* strain during mitosis. (**A**) Time-lapse images of the actin ring in the wt and *rpl1001Δ* strains during whole mitosis. Each frame corresponds to a 90 s interval. (**B**) Actin ring dynamics curve in the wt and *rpl1001Δ* strains during cell mitosis (*n* = 30), dotted lines indicate transition from formation to the contraction period of actin ring. (**C**) Actin ring length of the wt and *rpl1001Δ* strains during mitosis (*n* = 30). (**D**) Actin ring formation and contraction time of the wt and *rpl1001Δ* strains during mitosis (*n* = 30). (**E**) Actin ring contraction rate of the wt and *rpl1001Δ* strains (*n* = 30). The black dots indicate outliers. Pact1-LifeAct-mGFP, actin-LifeAct-mutant green fluorescent protein. ** *p* < 0.01.

**Figure 7:**
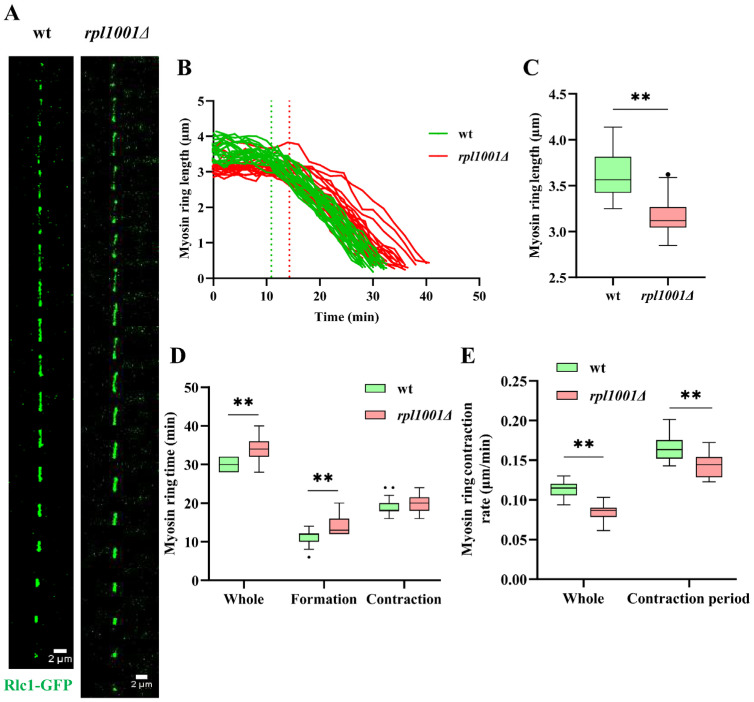
Changes of myosin ring in the *rpl1001Δ* strain during mitosis. (**A**) Time-lapse images of myosin ring in the wt and *rpl1001Δ* strains during whole mitosis. Each frame corresponded to 120 s interval. (**B**) Myosin ring dynamics curve in the wt and *rpl1001Δ* strains during cell mitosis (*n* = 20), dotted lines indicate transition from formation to the contraction period of myosin ring. (**C**) Myosin ring length in the wt and *rpl1001Δ* strains during mitosis (*n* = 20). (**D**) Myosin ring formation and contraction time of the wt and *rpl1001Δ* strains (*n* = 20). (**E**) Myosin ring contraction rate of the wt and *rpl1001Δ* strains (*n* = 20). The black dots indicate outliers. Rlc1-GFP, myosin II regulatory light chain-green fluorescent protein. ** *p* < 0.01.

During mitosis, myosin-II and F-actin assemble into shrunken rings along the cell equatorial plate to aid cell division. Time-lapse imaging of cells showed that actin and myosin gradually aggregated during anaphase of mitosis to form a contractile ring, which began to contract and finally degraded upon completion of its assembly (Figure 6A and Figure 7A). The length of the actin ring was 3.7 ± 0.3 and 3.6 ± 0.4 μm for the wild-type and *rpl1001Δ* strains, respectively, the total time from actin ring formation to disappearance was 29.6 ± 3.1 and 31.9 ± 5.6 min, respectively, and the total contraction rate of the actin ring was 0.1 μm/min for both, with no significant differences between both groups. Further statistics on the actin ring assembly process revealed that the actin ring assembly times of the wild-type and the *rpl1001Δ* strains were 12.9 ± 2.7 and 15.4 ± 2.5 min, respectively, with significant differences. But upon entering the contraction phase, the contraction times of the wild-type and *rpl1001Δ* strains were 16.8 ± 3.2 and 16.5 ± 4.6 min, respectively, and the contraction rates were 0.2 μm/min for both (Figure 6B–E), which were not significantly different. The above results suggested that the *rpl1001* gene deletion prolongs actin ring assembly.

Statistical analysis of the myosin rings showed that the lengths of the myosin rings were 3.6 ± 0.3 and 3.2 ± 0.2 μm in the wild-type and *rpl1001Δ* strains, respectively, the total times for myosin ring formation to disappearance were 30.1 ± 1.7 and 34.0 ± 3.2 min, respectively, which were all significantly different. The total contraction rate of the myosin ring of the *rpl1001Δ* strain was also found to significantly slow down. Further statistics on the myosin ring assembly process revealed that the myosin ring assembly times were 10.9 ± 2.1 and 14.2 ± 2.6 min for the wild-type and *rpl1001Δ* strains, respectively, and also significantly different. Upon entering the contraction phase, the myosin rings contraction times of the wild-type and *rpl1001Δ* strains were 19.2 ± 2.4 and 19.8 ± 2.4 min, and the contraction rates were 0.2 and 0.1 μm/min, respectively (Figure 7B–E). The contraction time was not significantly different in the *rpl1001Δ* strain compared to the wild-type, but the rate of contraction was significantly lower. The above results indicated that the *rpl1001* gene deletion resulted in a shorter total length of myosin ring, a decrease in the rate of contraction, and an increase in the duration of its presence.

### 3.6. Mitochondrial Changes in the rpl1001Δ Strain

**Figure 8:**
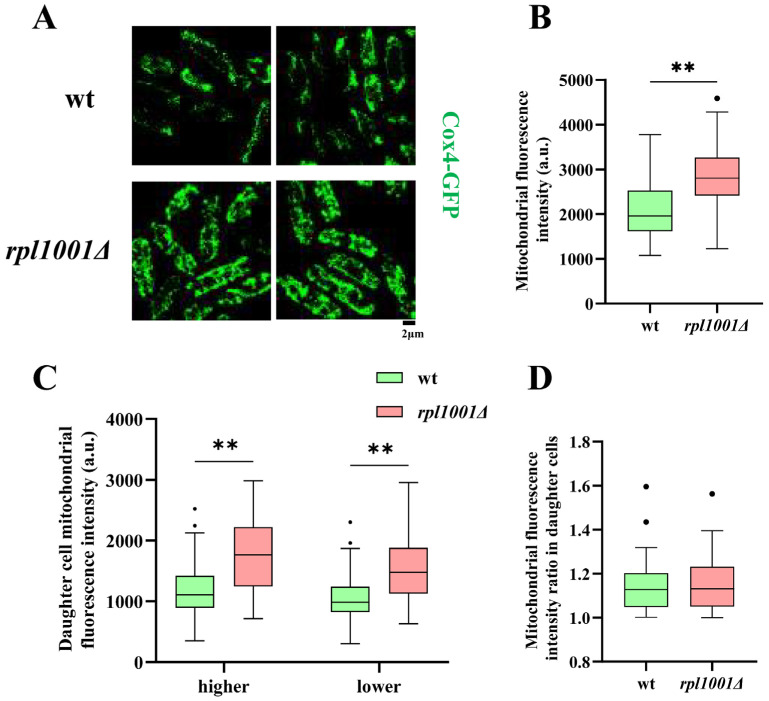
Mitochondrial changes in the *rpl1001Δ* strain. (**A**) Morphological images of mitochondria in the wt and *rpl1001Δ* strains. (**B**) Mitochondrial fluorescence intensity of the wt and *rpl1001Δ* strains (*n* = 60). (**C**) Mitochondrial fluorescence intensity of daughter cells in the wt and *rpl1001Δ* strains (*n* = 60). (**D**) Ratio of mitochondrial fluorescence intensity of daughter cells in the wt and *rpl1001Δ* strains (*n* = 60). Mitochondrial fluorescence intensity ratio in daughter cells represents the ratio of the higher mitochondrial fluorescence intensity value to the lower mitochondrial fluorescence intensity value in the two daughter cells obtained after cell division. The black dots indicate outliers. Cox4-GFP, cytochrome coxidase 4-green fluorescent protein. ** *p* < 0.01.

Mitochondria play the role of energy factories in the cell, and they regulate the process of cell division through their fission and fusion. The statistical results of mitochondria fluorescence intensity showed that they were 2081.3 ± 644.7 and 2829.2 ± 735.4 a.u. in the wild-type and *rpl1001Δ* strains, respectively, which were significantly different (Figure 8A,B), indicating that *rpl1001* gene deletion could increase the mitochondrial content.

As the cell undergoes division, the mitochondria split into the daughter cells. Statistics on the fluorescence intensity of subcellular mitochondria revealed that the average fluorescence intensity of subcells with more mitochondria in the wild-type strain was 1188.9 ± 455.2 a.u., and that of subcells with fewer mitochondria was 1047.5 ± 399.9 a.u.; and the average fluorescence intensities in the *rpl1001Δ* strain was 1766.0 ± 592.8 and 1542.3 ± 518.4 a.u., respectively (Figure 8C,D). The fluorescence intensity of both daughter cells showed a highly significant difference among the wild-type and the *rpl1001Δ* strains, suggesting that the *rpl1001* gene deletion also increased the mitochondrial content of daughter cells, but had no effect on the proportion of mitochondria distributed among the daughter cells during mitosis.

### 3.7. Analysis of Highly Expressed Genes and Differentially Expressed Genes

**Figure 9:**
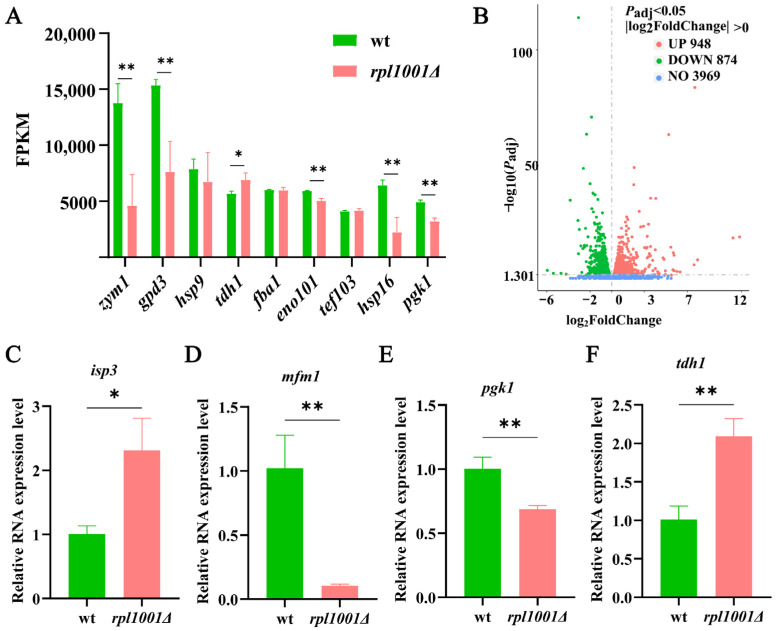
Analysis of highly expressed genes and differentially expressed genes, and qRT-PCR verification of key differentially expressed genes. (**A**) Differential analysis of highly expressed genes in the wt and *rpl1001Δ* strains. (**B**) Volcano plot of differently expressed genes in the wt and *rpl1001Δ* strains. (**C**–**F**) Relative mRNA expression of *isp3*, *mfm1*, *pgk1*, and *tdh1* measured by qRT-PCR in the wt and *rpl1001Δ* strains. * *p* < 0.05, ** *p* < 0.01.

The transcriptome sequencing analysis of the wild-type and *rpl1001Δ* strains was performed, and their gene expression levels were quantified after quality control analysis (Table S1). FPKM > 4000 means extremely highly expressed genes, FPKM > 60 means highly expressed genes, FPKM > 1 means expressed genes, and FPKM < 1 means lowly expressed genes or non-expressed genes. The investigations revealed that the wild-type strain had 4951 expressed genes and 1967 high-level expressed genes, while the *rpl1001Δ* strain had 5039 expressed genes and 1959 high-level expressed genes (Table 3, Figure 9A).

Among the genes that the *rpl1001Δ* and wild-type strains both express to extremely high levels, *tdh1* encodes glyceraldehyde-3-phosphate dehydrogenase, Tdh1, which physically binds to response regulators (Mcs4) and stress-responsive MAPKKKs, and are involved in the positive regulation of the MAPK pathway. Through phosphorylation, the MAPK pathway delivers signaling molecules that regulate biological processes including cell growth, division, and differentiation [23]. The FPKM value of *tdh1* was upregulated in the *rpl1001Δ* strain, indicating that the cellular phosphorylation of MAPK signaling pathway was compensated. The *zym1* gene encodes a metallothionein, Zym1, and deficiency of Zym1 leads to abnormal chromosome segregation in meiosis and abnormal spore formation [24,25]. The FPKM of the *zym1* gene was greatly reduced in the *rpl1001Δ* strain, which is consistent with the abnormal spore production number in this study.

Further investigation showed that only the wild-type strain appeared to express *hsp16* and *pgk1* at extremely high levels. The *hsp16* gene encodes the heat shock protein, Hsp16, a Hsp family protein that regulates cell cycle checkpoints under stress [26]. The *pgk1* gene encodes phosphoglycerate kinase, Pgk1, affecting mitotic cell cycle progression [27]. The FPKM values of *hsp16* and *pgk1* were greatly reduced in the *rpl1001Δ* strain, indicating abnormal cell cycle progression, which is consistent with the statistics of abnormal cell division in the *rpl1001Δ* strain.

The differentially expressed genes (DEGs) were analyzed in the *rpl1001Δ* and wild-type strains, and *P*_adj_ < 0.05 and |log_2_FoldChange| > 0 were set as the screening criteria. The results showed that compared to the wild-type, there were 1822 differentially expressed genes in the *rpl1001Δ* strain, including 874 downregulated genes and 948 upregulated genes (Figure 9B).

Among the up-regulated genes (Table 4), *mat2-Pi* gene encodes the protein that is involved in activating the mitotic to meiotic cycle switch by binding DNA transcription factors with a specific structure [28]. Meanwhile, *ftm4* encodes for the sub-telomeric 5Tm protein family, Ftm4, and *Tf2-13* encodes for the retrotransposable element/transposon Tf2-type. During amino acid starvation in cells, the expression levels of *ftm4* and *Tf2-13* genes are upregulated to regulate nucleic acid levels [29]. The protein expressed by *isp3* is a structural component of the spore wall and is resistant to nutrient-deficient environments [30]. In the *rpl1001Δ* strain, *mat2-Pi*, *Tf2-13*, *ftm4*, and *isp3* gene expression was upregulated, suggesting that the deletion of *rpl1001* increased intracellular RNA levels to initiate a transcriptional program to resist environmental change when cells are in a similarly nutrient-deficient environment.

Among the down-regulated genes (Table 5), *mfm1* and *mfm2* encode the M-factor precursor, which is involved in cell-cell signaling and regulates the MAPK cascade response in sporulation [31]. The *pcm2* gene encodes protein-l-isoaspartate o-methyltransferase Pcm2, which affects spore formation [24]. The *dis1* gene encodes TOG/XMAP215 microtubule plus end tracking polymerase, Dis1, involved in microtubule formation, depolymerization, shortening, and elongation [32]. Down-regulated expression of *mfm1*, *mfm2*, *pcm2*, and *dis1* genes by 3.1, 2.5, 2.3, and 2.0 fold, respectively, occurred in the *rpl1001Δ* strain. The results suggest that the deletion of *rpl1001* hindered the formation and contraction of cellular MTs, and led to abnormal division of spore nucleus in meiotic process, which affected the production of spores. This was consistent with the phenotype of microtubule abnormalities and a significant increase in spore numbers in the *rpl1001Δ* strain.

### 3.8. Differentially Expressed Gene Validation by qRT-PCR

According to the differential gene expression analysis and using *act1* as the reference gene, the key genes *isp3*, *mfm1*, *pgk1*, and *tdh1* were screened for qRT-PCR validation (Figure 9C–F). The results showed that *isp3* and *tdh1* were up-regulated and *mfm1* and *pgk1* were down-regulated in both RNA-seq and qRT-PCR. The expression trends of the above key genes after *rpl1001* deletion were consistent with the RNA-Seq results, indicating that the results of the transcriptome experiments were reliable.

### 3.9. Analysis of Differential Genes for GO and KEGG Enrichment

**Figure 10:**
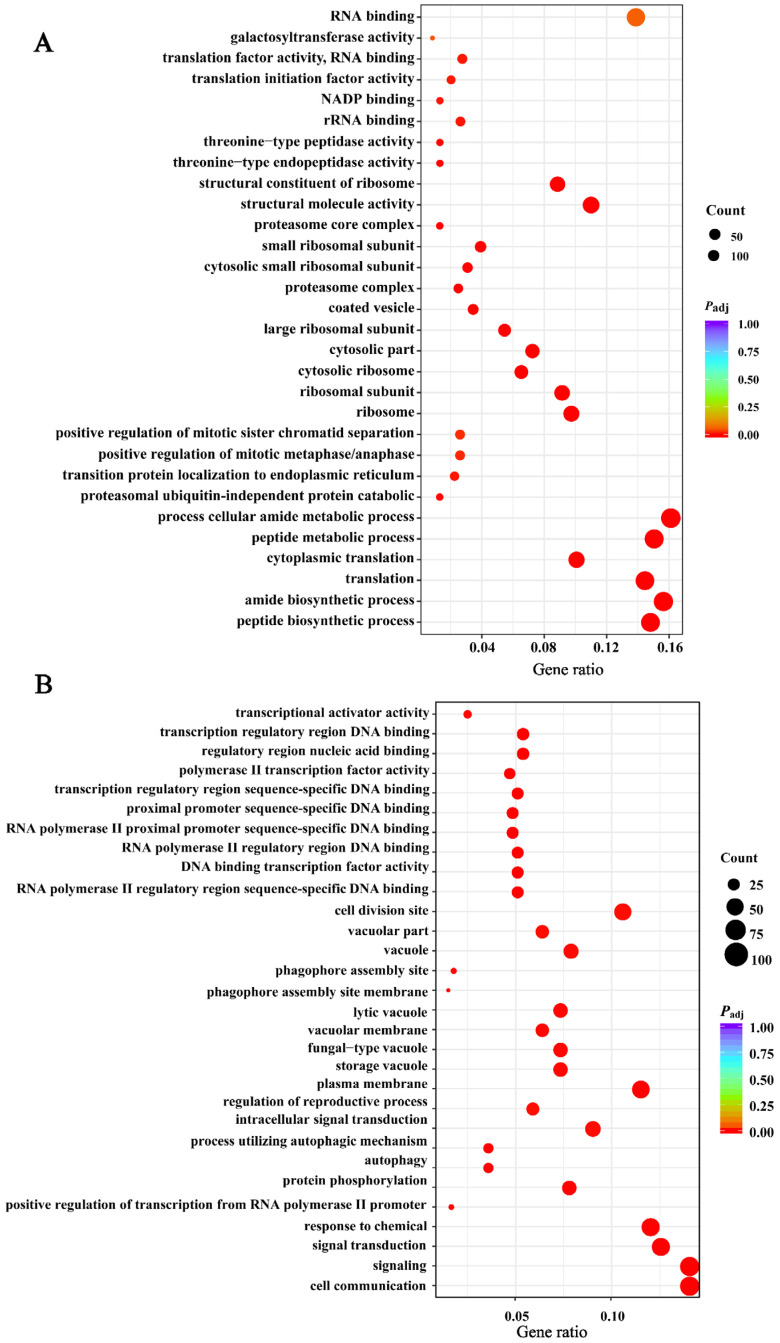
The GO enrichment results of differentially expressed genes in the wt and *rpl1001Δ* strains. (**A**) GO enrichment results for up-regulated differential genes. (**B**) GO enrichment results for down-regulated differential genes.

The differential genes were enriched to 196 GO clades in the *rpl1001Δ* strain, including 41 molecular functions, 97 biological processes, and 58 cellular components, compared with the wild-type strain. Listed in descending order of *P*_adj_ value, the top 10 up-regulated and down-regulated genes were analyzed in terms of their biological functions. The results showed that up-regulated genes were mainly enriched in peptide and amide synthesis and proteasome-mediated non-ubiquitin-dependent proteolytic metabolism in biological processes. In cellular components, up-regulated genes were mainly enriched in the ribosomal region. In molecular functions, up-regulated genes were mainly enriched in molecular structural activities and ribosomal structures (Figure 10A). Moreover, downregulated differential genes were enriched in cellular communication and autophagy processes, cytoplasmic membrane regions in cellular components, and transcriptional protein functions in molecular functions (Figure 10B). The GO enrichment results indicated that the ribosome biogenesis process of the *rpl1001Δ* strain was affected, that the intracellular protein formation and degradation process was destabilized, and the well-ordered order of the cell division process was disrupted.

**Figure 11:**
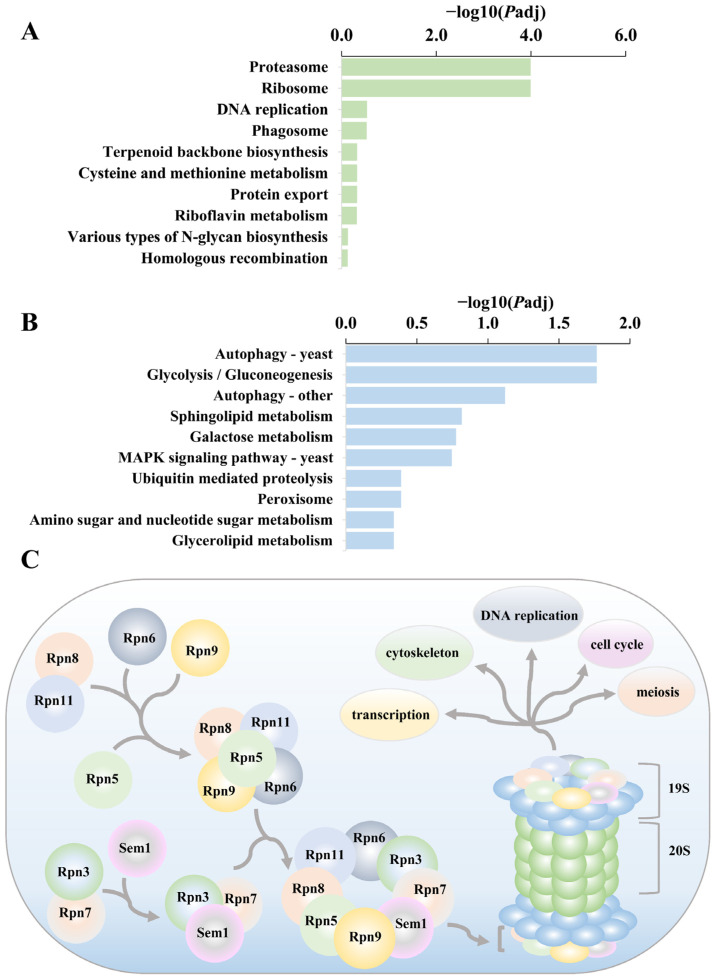
The KEGG enrichment results of differentially expressed genes in the wt and *rpl1001Δ* strains. (**A**) KEGG enrichment results for upregulated differential genes. (**B**) KEGG enrichment results for downregulated differential genes. (**C**) Schematic of the process of proteasome lid assembly and the process of proteasome regulation.

KEGG enrichment analysis of differential gene results showed 549 differential genes were enriched in 80 pathways in the *rpl1001Δ* strain compared to the wild-type strain. The top 10 pathways of both up-regulated genes and down-regulated genes were taken and analyzed by *P*_adj_ value (Figure 11A,B). Up-regulated genes were mainly concentrated in pathways like proteasome, ribosome and DNA replication, while down-regulated genes were enriched in pathways such as autophagy and glycolysis.

The proteasome is essential for protein and amino acid homeostasis, which controls the cell cycle, cytoskeleton, DNA replication, meiosis, or transcription [33]. In the proteasome pathway, 21 up-regulated genes were enriched (Figure 11A). In this pathway, the major genes whose expression was upregulated included *rpn5*, *rpn8*, *rpn11*, *rpn12*, and *rpt4*.

The regulatory subunit of the 19S proteasome is composed of proteins encoded by *rpn5*, *rpn8*, *rpn11*, *rpn12*, and *rpt4* genes, which belong to the Rpn family (Figure 11C). The deficiency of Rpn5 causes accumulation of polyubiquitinated proteins, exacerbates growth defects in proteasome mutants, and leads to abnormal cell mitosis [34]. The Rpn8 protein is localized to the spindle intermediate region through the importin alpha family nuclear import signal receptor adaptor, Imp1. Rpn8 possesses metallopeptidase activity and participates in the rapid degradation of proteins assembled in the spindle’s intermediate region at the end of mitosis for efficient mitotic spindle disassembly [35]. During mitosis and meiosis, Rpn11 protein accumulates mainly at the periphery of the nucleus, and during spore formation, it diffuses from the periphery of the nucleus to the cytoplasm and regulates spore development [36]. The Rpn12 protein regulates the degradation of ubiquitin conjugates and the deficiency of this protein will hinder the degradation of cell cycle regulators, leading to the stagnation of the transition from metaphase to anaphase while affecting the normal progress of the cell cycle [37]. The *rpt4* gene encodes 19S proteasome base subcomplex ATPase subunit Rpt4, which binds and hydrolyzes ATP to provide energy for meiosis. In the *rpt4Δ* strain, the mating efficiency and the formation of spores were decreased [25]. The abnormal expression of proteasome pathway genes suggested that cell division was affected in the *rpl1001Δ* strain, which was consistent with the abnormalities in sporulation and mitosis in the *rpl1001Δ* strain, and also consistent with the results of the GO enrichment analysis (Figure 10).

## 4. Discussion

The modification of the spindle polar body (SPB), assembly of forespore membrane (FSM), and formation of spore wall regulate spore formation in fission yeast [38]. The spore wall structure protein, Isp3, accumulates in the cytoplasm of the forespore and is then exported to the forespore membrane to form the outermost layer of the spore wall [30]. The anaphase-promoting complex/cyclosome (APC/C) activator, Fzr1, is a key factor in the termination of meiosis and is also involved in promoting the coordination of spore wall formation, such that more than four spores will be produced in Fzr1-deficient strains [39]. The microtubule plus-end binding protein, Mal3, belongs to the EB1 family. A Mal3 deficiency affects meiosis and spore formation, and *mal3Δ* strains appear to produce more than four (up to eight) spores [40]. The mating pheromone M-factor precursor is formed by the *mfm1* and *mfm2* genes. The pheromone is recognized by sexually opposite, specific receptors on the surface of mating cells and stimulates heterotrimeric G proteins associated with the receptors, which in turn activates the MAPK signaling pathway and regulates spore formation [41]. Proteasomal activity is necessary for meiotic nuclear division [42]. During meiosis, proteasomes are dispersed on the nucleus and interact with the mitotic and meiotic cohesin loader subunit, Pds5, which regulates meiotic chromosome spindle length and influences chromosome segregation [43]. In this study, *isp3* gene expression was up-regulated and *mfm1* and *mfm2* gene expression was down-regulated in the *rpl1001Δ* strain, and KEGG enrichment analysis uncovered that 21 up-regulated genes in the proteasome pathway were significantly enriched. These key genes and pathways act in concert to regulate spore formation in meiosis, leading to a significant increase in the number of spores and the production of eight spores in the *rpl1001Δ* strain.

Upon entering division, microtubules, microtubule-associated proteins (MAPs), motor proteins, and other regulatory proteins form a dynamically structured bipolar spindle, which effectively captures sister chromatids. The microtubule-associated proteins organize the spindle microtubules into antiparallel microtubule overlap zones. Kinesin generates the force that separates the bipolar spindle, and the separation of the bipolar spindle in turn leads to the proper segregation of chromosomes [44,45]. Mitotic centromere-SPB clustering protein, Csi1, recruits the Alp7p-Alp14p protein complex into SPB and promotes bipolar spindle formation. After *csi1* gene deletion, the cells appeared to have a monopolar spindle, causing chromosome segregation defects [46]. The SPB cell cycle signalling scaffold, Cut12, is localized in the SPB throughout the cell cycle. Monopolar spindles formed in the *cut12* mutant, which led to failed chromosome segregation, and fatal cytokinesis [47]. Dis1, a protein member of the XMAP215/TOG family, is localized to mitoplasts during cytokinesis and is dependent on phosphorylation by cyclin-dependent protein kinase (Cdc2). During mitosis, Dis1 targets the kinesin-8 family plus-end directed microtubule motor, Klp5, to the centromere and participates in chromosome separation [48,49,50]. In this study, it was found that after the *rpl1001Δ* strain entered mitosis, the formation of bipolar spindles decreased significantly, while the proportion of monopolar spindles increased. It was also found that *dis1* gene expression was down-regulated in the *rpl1001Δ* strain. The result indicates that the spindle was unable to generate a balanced force to push itself to segregate correctly, and the attachment process of the spindle-targeted centromere is blocked, which leads to the phenotype of uneven chromosome segregation.

Myosin II and F-actin assemble into contractile rings along the equatorial plate of the cell, and myosin undergoes ATP hydrolysis to promote the movement of actin and drive the contraction of the ring to achieve cell division [51]. During mitosis, ATP consumption is required for the formation of both actin and myosin rings [52]. When phosphorylation of myosin II regulatory light chain, Rlc1, was inhibited, myosin II heavy chain (Myo2) activity was also reduced, resulting in a significant prolongation of myosin ring assembly [53]. The paxillin-like protein, Pxl1, is a component of the myosin ring, and in the absence of Pxl1, the contraction rate of the myosin ring is significantly slower [54]. Alpha-actinin (Ain1) has molecular articulator activity, which maintains the actin cytoskeleton and participates in the assembly of actin filament bundles. In the absence of Ain1 protein, the formation of the actin ring was delayed and the formation time of theactin ring was increased [55]. Heat shock protein, Hsp16, is a related protein that stabilize the contractile ring structure composed of myosin-II and F-actin [56]. In this study, it was found that the deletion of *rpl1001* gene caused the down-regulation of *hsp16* and *pgk1* gene expression which indicates that the structural stability of the actin-myosin ring was affected and the phosphorylation of intracellular phosphoglycerate and glycolysis were blocked, consistent with the phenotype of the prolonged assembly time of the actin and myosin rings.

## 5. Conclusions

This study revealed that the deletion of the *rpl1001* gene caused slow cell growth, increased cell volume, elicited an abnormal number of spores, distorted microtubule dynamics, unevenly affected chromosome separation, delayed formation of actin-myosin contraction ring, and increased mitochondrial content in fission yeast cells. Analysis of the RNA-Seq sequencing results showed that the proteasome pathway, upregulation of *isp3*, and downregulation of *mfm1* and *mfm2* in the *rpl1001Δ* strain were the main factors underlying the increased number of spores. Down-regulation of *dis1* caused the abnormal microtubule and chromosome dynamics, and down-regulation of *hsp16* and *pgk1* were the key genes affecting the delay of actin ring and myosin ring formation. The transcriptome data were reliable and the expression pattern of key differentially regulated genes after *rpl1001* deletion was consistent with the RNA-Seq results. Altogether, the results revealed the cell cycle changes and molecular mechanisms of the *rpl1001Δ* strain, which shed light on the function on the Rpl1001 protein during cell division.

## Figures and Tables

**Table 1:** List of experimental strains.

Strains	Genotypes	Source
PT286	*h- wt ade6-210 leu1-32 ura4-D18*	Lab. Trans ^1^
PT287	*h+ wt ade6-210 leu1-32 ura4-D18*	Lab. Trans
PT4436	*h- Hht1-RFP-KanR GFP-Atb2-KanR ade6-210 leu1-32 ura4-D18*	Lab. Trans
PT3850	*h+ Pact1-LifeAct-mGFP::leu1*	Lab. Trans
PT2006	*h- Rlc1-GFP::ura4 leu1-32*	Lab. Trans
YL18	*h- mCherry-Atb2-HygR Cox4-GFP::leu2 ade6-210 leu1-32 ura4-D18*	This study
2599-1	*h+ rpl1001Δ::kanR*	This study
2599-2	*h- rpl1001Δ::kanR*	This study
2599-3	*h* *? rpl1001Δ::kanR Hht1-RFP-KanR GFP-Atb2-KanR ade6-210 leu1-32 ura4-D18*	This study
2599-4	*h? rpl1001Δ:: kanR Pact1-LifeAct-mGFP::leu1*	This study
2599-5	*h? rpl1001Δ:: kanR Rlc1-GFP::ura4*	This study
2599-6	*h? rpl1001Δ:: kanR mCherry-Atb2-HygR Cox4-GFP::leu2 ade6-210 leu1-32 ura4-D18*	This study

^1^ Laboratory of Associate Professor Phong Tran.

**Table 2:** Primer sequences used for qRT-PCR.

Gene	Forward Primer (5′→3′)	Reverse Primer (5′→3′)
*isp3*	TTT GGT GAT GGT GAC TGC GA	GAG TGG ATC AGG GCA GGT TC
*mfm1*	TG GAC TCA ATG GCT AAC TCC G	A ACG TAG GCA AGA AAA GTG GC
*pgk1*	C TTG AAG CCT GTT GCT GCT G	TC CTC GAT GTG GAA ACG CAA
*tdh1*	TC GTC AAG CTC GTC TCT TGG	G CAT TGC CTT TAA GCA CCC A
*act1*	CCC AAA TCC AAC CGT GAG AAG	CC AGA GTC CAA GAC GAT ACC AGT G

**Table 3:** Highly expressed genes in the wt and *rpl1001Δ* strains.

Gene	FPKM (wt)	FPKM (*rpl1001Δ*)	Description
*zym1*	13,760.9	4601.6	Metallothionein, Zym1
*gpd3*	15,353.5	6102.4	Glyceraldehyde 3-phosphate dehydrogenase, Gpd3
*hsp9*	7879.5	7169.7	Heat shock protein, Hsp9
*fba1*	6012.5	6445.7	Fructose-bisphosphate aldolase, Fba1
*tdh1*	5679.2	6817.2	Glyceraldehyde-3-phosphate dehydrogenase, Tdh1
*eno101*	5927.2	5517.0	Enolase
*tef103*	4107.5	4608.1	Translation elongation factor EF-1 alpha Ef1a-c
*hsp16*	6429.3	3209.0	Heat shock protein, Hsp16
*pgk1*	4919.3	2722.2	Phosphoglycerate kinase, Pgk1

**Table 4:** Upregulated DEGs in the wt and *rpl1001Δ* strains.

Gene	log_2_ Fold Change	*P*_adj_	Description
*mat2-Pc*	11.8	1.3 × 10^−18^	Silenced P-specific polypeptide Pc
*Tf2-13*	7.9	1.4 × 10^−8^	Retrotransposable element/transposon Tf2-type
*mat2-Pi*	7.6	2.2 × 10^−6^	Silenced P-specific polypeptide Pi
*aim27*	5.8	7.4 × 10^−4^	ER membrane protein complex subunit Aim27
*pdc102*	5.8	1.4 × 10^−10^	Pyruvate decarboxylase
*str3*	5.7	1.2 × 10^−11^	Siderophore-iron transporter Str3
*ftm4*	5.3	1.6 × 10^−63^	*S. pombe* specific 5Tm protein family
*shu1*	4.7	2.0 × 10^−9^	Heme import protein Shu1
*isp3*	4.0	5.4 × 10^−13^	*Schizosaccharomyces pombe* specific protein Isp3
*fmp40*	3.8	4.3 × 10^−2^	UPF0061 family mitochondrial protein

**Table 5:** Downregulated DEGs in the wt and *rpl1001Δ* strains.

Gene	log_2_ Fold Change	*P*_adj_	Description
*mat3-Mc*	−6.0	6.8 × 10^−5^	Mating-type m-specific HMG-box transcription factor Mc at silenced MAT3 locus
*mfm1*	−3.1	3.3 × 10^−28^	M-factor precursor Mfm1
*mfm2*	−2.5	9.2 × 10^−17^	M-factor precursor Mfm2
*mam1*	−2.4	1.5 × 10^−24^	M-factor transmembrane transporter Mam1
*mel1*	−2.4	4.7 × 10^−9^	Alpha-galactosidase, melibiase
*pcm2*	−2.3	7.8 × 10^−67^	Protein-L-isoaspartate O-methyltransferase Pcm2
*ftm7*	−2.2	2.7 × 10^−3^	*S. pombe* specific 5Tm protein family
*hry1*	−2.1	4.6 × 10^−6^	HHE domain cation binding protein
*arg4*	−2.1	1.4 × 10^−3^	Arginine specific carbamoyl-phosphate synthase Arg4
*dis1*	−2.0	8.2 × 10^−45^	TOG/XMAP14 microtubule-associated protein Dis1

## Data Availability

Publicly available datasets were analyzed in this study.

## Supplementary Materials

The following supporting information can be downloaded at: https://www.mdpi.com/article/10.3390/cimb46030164/s1, Table S1: Statistics of the transcriptome sequencing quality analysis of the wt and *rpl1001Δ* strains.
