# Supplementary material for: Effects of rpl1001 Gene Deletion on Cell Division of Fission Yeast and Its Molecular Mechanism
Source: Curr Issues Mol Biol. 2024 Mar 18;46(3):2576–97. doi: 10.3390/cimb46030164 (PMC10969133; doi:10.3390/cimb46030164)
Supplement: Supplementary file 1 [file cimb-46-00164-s001.zip › cimb-2843431-supplementary.pdf]

Table S1. Statistics of transcriptome sequencing quality analysis of the wt and *rpl1001Δ* strains

Sample	Raw reads	Clean reads	Clean bases (G)	Error rate	Q20%	Q30%	GC pct
wt-1	47703748	46447832	7.0	0.0	98.3	94.6	41.4
wt-2	46164428	45173776	6.8	0.0	98.5	95.1	41.4
wt-3	45435808	44388600	6.7	0.0	98.1	94.1	41.2
rpl1001-1	46569660	45398452	6.8	0.0	98.6	95.5	41.7
rpl1001-2	45874544	44824696	6.7	0.0	98.4	94.9	41.5
rpl1001-3	47331844	46056410	6.9	0.0	98.3	94.8	41.5
